# Immunohistochemical Characterisation of Mismatch Repair Proteins in Feline Cancers: A Pilot Study Using Validated Cross‐Reactive Antibodies

**DOI:** 10.1111/vco.70105

**Published:** 2026-08-16

**Authors:** Shoma Nishibori, Masashi Sakurai, Yumiko Kagawa, Kazuo Nishigaki, Takayuki Nakagawa, Sakuya Inanaga, Masaya Igase, Takuya Mizuno

**Affiliations:** ^1^ Laboratory of Molecular Diagnostics and Therapeutics, Joint Graduate School of Veterinary Medicine Yamaguchi University Yamaguchi Yamaguchi Japan; ^2^ Laboratory of Veterinary Pathology, Joint Faculty of Veterinary Medicine Yamaguchi University Yamaguchi Yamaguchi Japan; ^3^ Division of Translational Research for One Medicine, Research Institute for Cell Design Medical Science Yamaguchi University Yamaguchi Yamaguchi Japan; ^4^ North Lab. Sapporo Hokkaido Japan; ^5^ Laboratory of Molecular Immunology and Infectious Disease, Joint Faculty of Veterinary Medicine Yamaguchi University Yamaguchi Yamaguchi Japan; ^6^ Laboratory of Veterinary Surgery, Graduate School of Agricultural and Life Sciences The University of Tokyo Tokyo Japan; ^7^ Japan Small Animal Cancer Center, Affiliated With the Japan Small Animal Medical Center Foundation Tokorozawa Saitama Japan

**Keywords:** cross‐reactive antibody, feline tumour, immune biomarkers, immunohistochemistry, mismatch repair

## Abstract

DNA mismatch repair (MMR) deficiency is a clinically important biomarker in human oncology, yet its relevance in feline neoplasia remains poorly understood due to limited characterisation and the absence of validated reagents. In this study, we established a practical immunohistochemistry (IHC) approach for evaluating feline MMR proteins by confirming the cross‐reactivity of anti‐human MLH1, MSH2 and MSH6 antibodies using CRISPR/Cas9‐engineered feline tumour cell lines lacking each target gene. Using these validated antibodies, we conducted a pilot screening to assess MMR protein expression in 83 feline tumours and observed variable reductions or losses of MLH1, MSH2 and MSH6 across several tumour types. Notably, MLH1 expression was lost in all melanoma cases (6/6, 100%) and in most osteosarcomas (14/15, 93%), indicating that MMR perturbations may be particularly relevant in these malignancies. These findings provide the first analytically validated framework for assessing MMR proteins in feline oncology and suggest that MMR dysregulation may be more widespread than previously recognised. Although the clinical sample size is limited, this initial pilot screening maps the preliminary landscape of feline MMR protein expression. Further large‐scale validation studies incorporating microsatellite instability testing, methylation analysis and genomic profiling are warranted to clarify the biological significance of these alterations.

## 1 Introduction

The DNA mismatch repair (MMR) system recognises and corrects base–base mismatches and insertion–deletion loops generated during DNA replication. In eukaryotes, mismatch recognition is mediated by the MutSα complex, composed of MutS homologue 2 (MSH2) and MutS homologue 6 (MSH6), whereas downstream repair involves the MutLα complex, consisting of MutL homologue 1 (MLH1) and postmeiotic segregation increased 2 (PMS2) [1]. Together, these complexes maintain replication fidelity; their disruption causes the accumulation of replication errors and microsatellite instability (MSI), resulting in high mutational burden. Beyond maintaining genomic stability, mismatch repair deficiency (dMMR) increases neoantigen load and remodels the tumour immune microenvironment, which in turn can confer sensitivity to immune checkpoint inhibition [2, 3, 4].

In human oncology, immunohistochemistry (IHC) for MLH1, MSH2, MSH6 and PMS2 is an integral component of the diagnostic workflow for identifying dMMR/MSI‐H tumours across cancer types, often used in combination with MSI testing to guide clinical decisions [5]. Consistent with their heightened immunogenicity, MSI‐H/dMMR tumours show marked responsiveness to immune checkpoint inhibitors, establishing MMR IHC as a clinically actionable predictive biomarker [6, 7].

In contrast, the MMR landscape in veterinary oncology—particularly in feline malignancies—remains poorly defined. To date, only one study has evaluated MLH1, MSH2 and MSH6 expression in feline gastrointestinal lymphoma [8], and the presence and distribution of MMR abnormalities in other feline tumour types are largely unknown. The absence of validated, cross‐reactive antibodies has further impeded systematic evaluation of feline MMR status. Meanwhile, interest in feline immunotherapy is growing, and anti‐feline PD‐1 monoclonal antibody (1A1‐2) and a chimeric derivative (1A1‐2‐fIgG_1_) have been developed [9, 10], demonstrating augmented feline immune activation. However, predictive biomarkers analogous to human dMMR/MSI‐H tumours are not yet established in cats, underscoring the need to characterise MMR alterations across feline tumour types.

In this study, we screened MLH1, MSH2 and MSH6 expression across various feline tumour types to determine their tissue‐specific patterns and the baseline frequency of protein loss. To ensure analytical validity, we evaluated the specificity of commercially available human MMR antibodies by generating CRISPR/Cas9‐engineered feline tumour cell lines lacking MLH1, MSH2 or MSH6 and confirming the corresponding loss of protein expression. We hypothesised that a subset of feline tumours would exhibit loss of MMR proteins detectable by cross‐reactive antibodies and that the distribution of potential dMMR would vary by tumour type.

## 2 Materials and Methods

### 2.1 Cell Lines

The D10 complete medium (DMEM supplemented with 10% fetal bovine serum [FBS], 100 U/mL penicillin, 100 μg/mL streptomycin and 55 μM 2‐mercaptoethanol) was used to maintain the human kidney cell line HEK293T, the two non‐neoplastic feline cell lines—feline kidney cell line Crandell–Rees Feline Kidney (CRFK; [11]) and feline macrophage cell line fcwf‐4 [12]**—**as well as the feline T‐cell lymphoma cell line FeLV‐3281 and feline thymic lymphoma cell line 3201 [13]. Only the FeLV‐3281 cell line was maintained in D10 medium without 2‐mercaptoethanol. Additionally, the feline T‐cell lymphoma cell line FT‐1 [14], feline B‐cell lymphoma cell line MS4 [15], feline lymphoblastoid cell line FL‐4 [16], peripheral blood mononuclear cell–derived cell line FeT‐J [17] and feline mammary gland tumour cell lines FONp, FYMp, FONm, FKNp and FMCm [18] were cultured in R10 complete medium (RPMI‐1640 supplemented with 10% FBS, 100 U/mL penicillin, 100 μg/mL streptomycin and 55 μM 2‐mercaptoethanol). All cell lines were maintained at 37°C in a humidified incubator with 5% CO_2_. FeLV‐3281, MS4 and FeT‐J cell lines were kindly provided by Dr. Kazuo Nishigaki (Yamagucni University). CRFK, fcwf‐4, FT‐1 and FL‐4 were generously provided by Dr. Hajime Tsujimoto (The University of Tokyo). All feline mammary gland tumour cell lines were established by Dr. Takayuki Nakagawa (The University of Tokyo).

### 2.2 Lentiviral Transduction of Knockout Vectors

The primers used for constructing knockout vectors are listed in Table 1. For the preparation of feline MLH1, MSH2 and MSH6 knockout vectors, pairs of oligonucleotides (YTM2826/YTM2827 for MLH1, YTM2832/YTM2833 for MSH2 and YTM2834/YTM2835 for MSH6) were annealed and ligated into the lentiCRISPR v2 backbone to generate lentiCRISPR‐fMLH1‐1 #12, lentiCRISPR‐fMSH2‐2 #28 and lentiCRISPR‐fMSH6‐1 #13, respectively. The lentiCRISPR v2 plasmid was obtained from Dr. Feng Zhang (Addgene plasmid #52961; http://n2t.net/addgene:52961; RRID:Addgene_52 961). To produce lentiviruses expressing sgRNAs targeting each gene, HEK293T cells were seeded at 7.5 × 10^5^ cells/well in six‐well plates 1 day before transfection. On the following day, 1.25 μg of each plasmid was mixed with PEI Max (Polysciences Inc., Warrington, PA, USA) in Opti‐MEM medium, incubated for 15 min at room temperature, and added to the cells. Twenty‐four hours after transfection, the medium was replaced with antibiotic‐free RPMI‐1640 complete medium. After another 24 h, the supernatant containing lentiviruses was collected and applied to FYMp cells. Twelve hours after infection, the supernatant was replaced with fresh medium and puromycin (2 μg/mL; InvivoGen, San Diego, CA, USA) was added to select stably transduced cells (FYMp/ko‐fMSH2 and FYMp/ko‐fMSH6). For FYMp/ko‐fMLH1, single‐cell clones were isolated by limiting dilution and used for subsequent western blot analysis.

**TABLE 1:** The primers used for construction of plasmid vectors.

Primer name	Direction	Nucleotide sequences (5′ to 3′)
YTM2826	F	CACCG‐agcgggggttattcggcggc
YTM2827	R	AAAC‐gccgccgaataacccccgct‐c
YTM2832	F	CACCG‐acctgttccatatgtacgac
YTM2833	R	AAAC‐gtcgtacatatggaacaggt
YTM2834	F	CACCG‐gggctataaagtagcacgag
YTM2835	R	AAAC‐ctcgtgctactttatagccc‐c

### 2.3 Western Blotting

Cells were lysed in NP‐40 lysis buffer (1% NP40, 10 mM Tris‐HCl [pH 7.5], 150 mM NaCl, 1 mM EDTA, protease inhibitor cocktails [Nacalai Tesque, Kyoto, Japan], 1 mM Na3VO4 and 50 mM NaF). Equal amounts of total protein (20 μg) were separated by SDS‐PAGE on 8% polyacrylamide gels and transferred onto PVDF membranes (Merck Millipore, Darmstadt, Germany). Membranes were blocked with 5% skimmed milk in TBS‐T and incubated overnight at 4°C with primary antibodies (the same as used for IHC) diluted 1:1000. After washing, membranes were incubated with horseradish peroxidase (HRP)‐conjugated secondary antibodies: anti‐mouse IgG (Thermo Fisher Scientific, Waltham, MA, USA; 1:10 000) or anti‐rabbit IgG (Jackson ImmunoResearch Inc., West Grove, PA, USA; 1:5000). Signals were detected using an Amersham ImageQuant 800 imager (Cytiva, Tokyo, Japan). To confirm equal protein loading, membranes were reprobed with a mouse monoclonal anti‐β‐actin antibody (AC‐74, Sigma‐Aldrich Japan KK, Tokyo, Japan).

### 2.4 IHC

Formalin‐fixed, paraffin‐embedded (FFPE) feline tumour samples were obtained from diagnostic pathology laboratories (North Lab). All samples were diagnosed by ACVP‐board‐certified veterinary pathologists. A total of 83 tumour samples were examined, including mammary carcinoma (7 cases), renal cell carcinoma (6), squamous cell carcinoma (20), fibrosarcoma (6), osteosarcoma (15), melanoma (6) and lymphoma (22). Additionally, to technically validate antibody specificity on the IHC platform, FFPE cell blocks were prepared from wild‐type and engineered MMR‐knockout feline cell lines. Briefly, cultured cells were harvested and washed twice with PBS. The cell pellets were fixed in 10% formalin at room temperature for 24–48 h. After removal of the formalin by centrifugation, the fixed cells were suspended in liquefied 2% agarose gel in PBS, centrifuged at 1200*g* for 7 min and solidified at 4°C for 30 min. The resulting cell‐containing gels were extracted and embedded in paraffin using standard histological procedures. Sections (4 μm thick) were mounted on charged MAS‐coated glass slides (Matsunami Glass Ind., Osaka, Japan). After deparaffinisation and rehydration, antigen retrieval was performed using Target Retrieval Solution, pH 9 (Agilent Technologies (Dako), Glostrup, Denmark) for MLH1 and MSH2 and 0.01 M citrate buffer (pH 6.0) for MSH6. Endogenous peroxidase activity was blocked with 3% hydrogen peroxide in methanol, and nonspecific binding was inhibited with 5% skimmed milk and 5% bovine serum albumin in PBS. The sections were incubated overnight at 4°C with the following primary antibodies: mouse monoclonal anti‐human MLH1 (clone G168‐15, Abcam, Cambridge, UK; 1:25), rabbit monoclonal anti‐human MSH2 (clone SP46, Abcam, Cambridge, UK; 1:50), mouse monoclonal anti‐human PMS2 (clone 163C1251, Novus Biologicals LLC, Centennial, CO, USA; 1:250), mouse monoclonal anti‐human PMS2 (clone 1G4E6, Proteintech Group Inc., Rosemont, IL, USA; 1:200) and mouse monoclonal anti‐human MSH6 (clone 44/MSH6, BD Transduction Laboratories, San Jose, CA, USA; 1:250). Isotype‐matched negative controls included anti‐rabbit IgG (clone DA1E, Cell Signalling Technology Inc., Danvers, MA, USA) and anti‐mouse IgG1 (clone P3.6.2.8.1, eBioscience Inc., San Diego, CA, USA). Sections were then incubated with Histofine Simple Stain MAX PO (M) or (R) kits (Nichirei Bioscience Inc., Tokyo, Japan), and immunoreactivity was visualised using the Peroxidase Stain DAB Kit (Nacalai Tesque Inc., Kyoto, Japan). After counterstaining with Mayer's haematoxylin, sections were dehydrated, cleared in xylene and mounted with xylene‐based mounting medium. For melanoma tissues, melanin bleaching was performed in 10% hydrogen peroxide in PBS for 10 min at 65°C before antigen retrieval. After incubation with the secondary antibody, the Simple Stain AEC Solution (Nichirei Bioscience Inc., Tokyo, Japan) was used instead of DAB. Finally, the sections were counterstained with Mayer's haematoxylin and mounted with water‐soluble mounting medium (Diagnostic BioSystems, Pleasanton, CA, USA). To confirm that the bleaching procedure did not abolish MMR immunoreactivity, the same bleaching condition was applied to positive‐control lymphoma sections, in which preserved nuclear staining for MLH1, MSH2 and MSH6 was confirmed.

Under the oversight of board‐certified veterinary pathologists, true expression ‘loss’ was defined as a complete absence of nuclear staining (0%) in tumour cells, provided that adjacent normal elements (e.g., stromal cells, lymphocytes or endothelial cells) within the same section showed robust nuclear staining as mandatory internal controls. This contrast between positive normal elements and negative tumour cells is representatively shown in a case of feline small intestinal lymphoma stained for MLH1 (Figure S1). Any specimen lacking reliable internal control staining was excluded from the analysis.

### 2.5 Ethical Approval and Consent

This retrospective study used anonymised, archived FFPE tissue specimens held by commercial veterinary diagnostic pathology laboratories. All specimens had originally been collected for routine diagnostic purposes, and only residual tissue remaining after completion of the diagnostic procedures was used. No samples were collected specifically for this study, and no additional procedures were performed on any animals. The researchers received no information that could identify individual animals or their owners. Therefore, this study did not involve the experimental use of live animals and did not require formal animal ethics committee approval. Specific informed owner consent for this study was not required because only fully anonymised residual diagnostic specimens were used.

## 3 Results

### 3.1 Cross‐Reactivity of Anti‐Human Monoclonal Antibodies With Feline MMR Proteins

To confirm that the commercially available anti‐human monoclonal antibodies used for IHC cross‐react with feline proteins, each feline MMR gene was disrupted in the feline mammary carcinoma cell line FYMp using the CRISPR–Cas9 system. First, Western blotting of lysates from these knockout cell lines demonstrated the loss of specific bands corresponding to MLH1, MSH2 and MSH6 when probed with the respective anti‐human monoclonal antibodies, confirming their cross‐reactivity with denatured feline proteins (Figure 1A). In addition, loss of MSH6 expression was observed in FYMp cells lacking MSH2, whereas MSH2 expression was markedly reduced in MSH6‐deficient FYMp cells.

**FIGURE 1:**
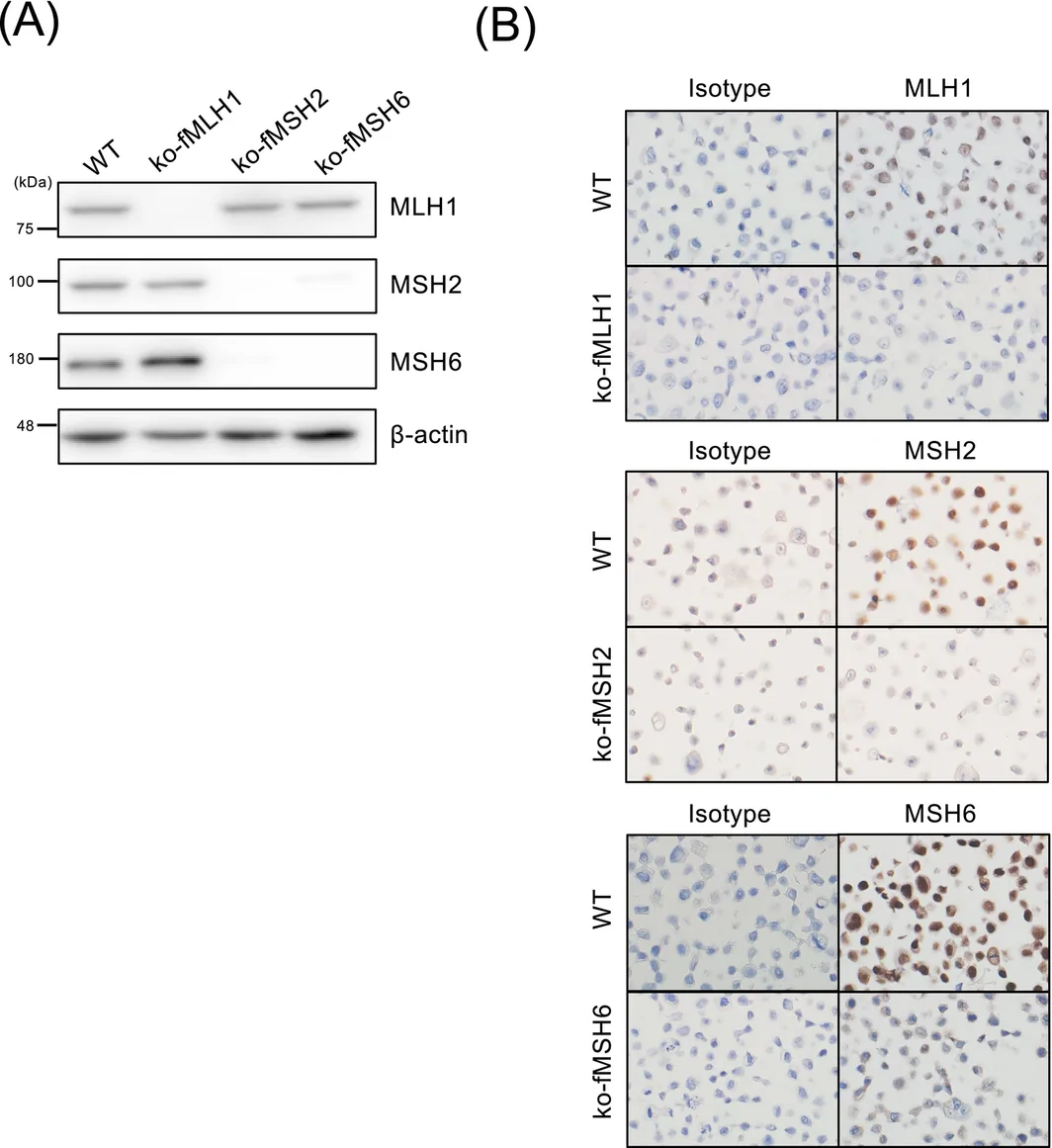
Validation of antibody specificity using CRISPR/Cas9‐engineered feline cell lines. (A) Western blot analysis of MMR proteins in wild‐type (WT) and MMR‐knockout (ko‐fMLH1, ko‐fMSH2 and ko‐fMSH6) feline mammary carcinoma cells (FYMp). Proteins were separated with SDS‐PAGE, followed by western blotting with anti‐human MLH1 antibody, anti‐human MSH2 antibody or anti‐human MSH6 antibody. These specific bands corresponding to each protein were detected in WT cells but were absent in the respective knockout lines, confirming the cross‐reactivity of the anti‐human monoclonal antibodies. β‐actin served as a loading control. A representative result of two independent experiments is shown. (B) Immunohistochemical analysis of MMR proteins using formalin‐fixed paraffin‐embedded (FFPE) cell blocks prepared from wild‐type (WT) and each MMR‐knockout feline cell line. Sections were incubated with the corresponding target‐specific primary antibody or its isotype‐matched control. Distinct nuclear immunolabelling is observed in the WT control cells, whereas nuclear signals are completely absent in all target‐disrupted knockout cell blocks.

To further validate antibody specificity under standard diagnostic IHC conditions, we performed immunohistochemical staining on FFPE cell blocks prepared from wild‐type and engineered MMR‐knockout feline cell lines. The IHC results demonstrated distinct nuclear immunolabelling in the respective wild‐type control cells, whereas nuclear signals were completely absent in all target‐disrupted knockout cell blocks (Figure 1B).

Finally, to confirm reactivity in native feline tissues, antibodies against MLH1, MSH2 and MSH6 were evaluated across normal feline tissues and showed distinct nuclear immunolabelling (representatively demonstrated in a feline mammary tumour metastasised to a lymph node; Figure S2). These three antibodies were therefore selected for subsequent analyses. In contrast, two different clones of commercially available anti‐human monoclonal antibody against PMS2 did not show nuclear staining and were excluded from the study.

### 3.2 Immunohistochemical Analysis of MMR Protein Expression in Various Feline Tumours

A total of 83 tumour samples from seven tumour types were examined (Table S1). Representative immunostaining patterns are shown in Figure 2. While some cases exhibited clear nuclear expression of MLH1, MSH2 and MSH6 in tumour cells (Figure 2A–C), others lacked expression of one or more proteins (Figure 2D–F). The numbers and percentages of negative cases for each MMR protein are summarised in Table 2 and Figure 3. MLH1 negativity was most frequent in melanomas (100%, 6/6), followed by osteosarcomas (93%, 14/15) and squamous cell carcinomas (45%, 9/20). MSH2 negativity was most common in osteosarcomas (73%, 11/15), followed by renal cell carcinomas (50%, 3/6) and melanomas (50%, 3/6). MSH6 negativity was observed most frequently in melanomas (33%, 2/6), followed by osteosarcomas (20%, 3/15) and renal cell carcinomas (17%, 1/6).

**FIGURE 2:**
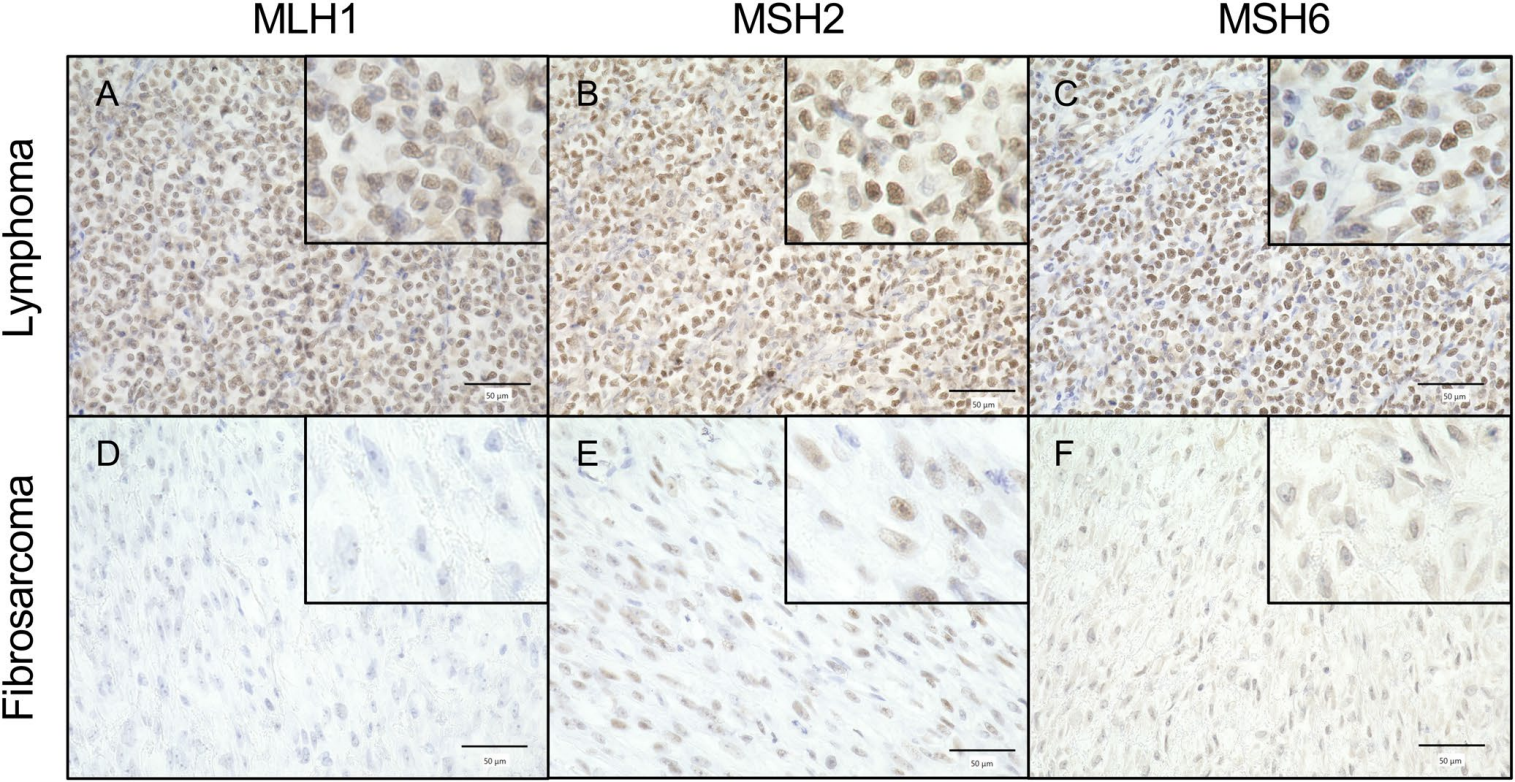
Representative immunohistochemical staining of mismatch repair (MMR) proteins in feline tumours. Representative images of feline lymphoma (A–C) and fibrosarcoma (D–F). (A–C) Lymphoma cells exhibiting positive nuclear staining for MLH1 (A), MSH2 (B) and MSH6 (C). (D–F) In the fibrosarcoma sample, MLH1 expression is lost (D), whereas nuclear staining for MSH2 (E) and MSH6 (F) is retained. The high‐magnification insets clearly demonstrate the presence or absence of clear nuclear immunoreactivity within the cells. Nuclei were counterstained with haematoxylin. Scale bars = 50 μm.

**TABLE 2:** Frequency of negative immunolabelling for MLH1, MSH2 and MSH6 in feline tumours.

Tumour type	Number of samples	Numbers of samples with negative immunolabelling		
		MLH1	MSH2	MSH6
Mammary carcinoma	7	2 (29%)	0 (0%)	0 (0%)
Renal cell carcinoma	6	1 (17%)	3 (50%)	1 (17%)
Squamous cell carcinoma	20	9 (45%)	5 (25%)	1 (5%)
Fibrosarcoma	6	1 (16%)	0 (0%)	0 (0%)
Osteosarcoma	15	14 (93%)	11 (73%)	3 (20%)
Malignant Melanoma	6	6 (100%)	3 (50%)	2 (33%)
Lymphoma	22	4 (18%)	5 (22%)	1 (5%)
Gastrointestinal	18	4 (22%)	5 (28%)	1 (6%)
Others	4	0 (0%)	0 (0%)	0 (0%)

**FIGURE 3:**
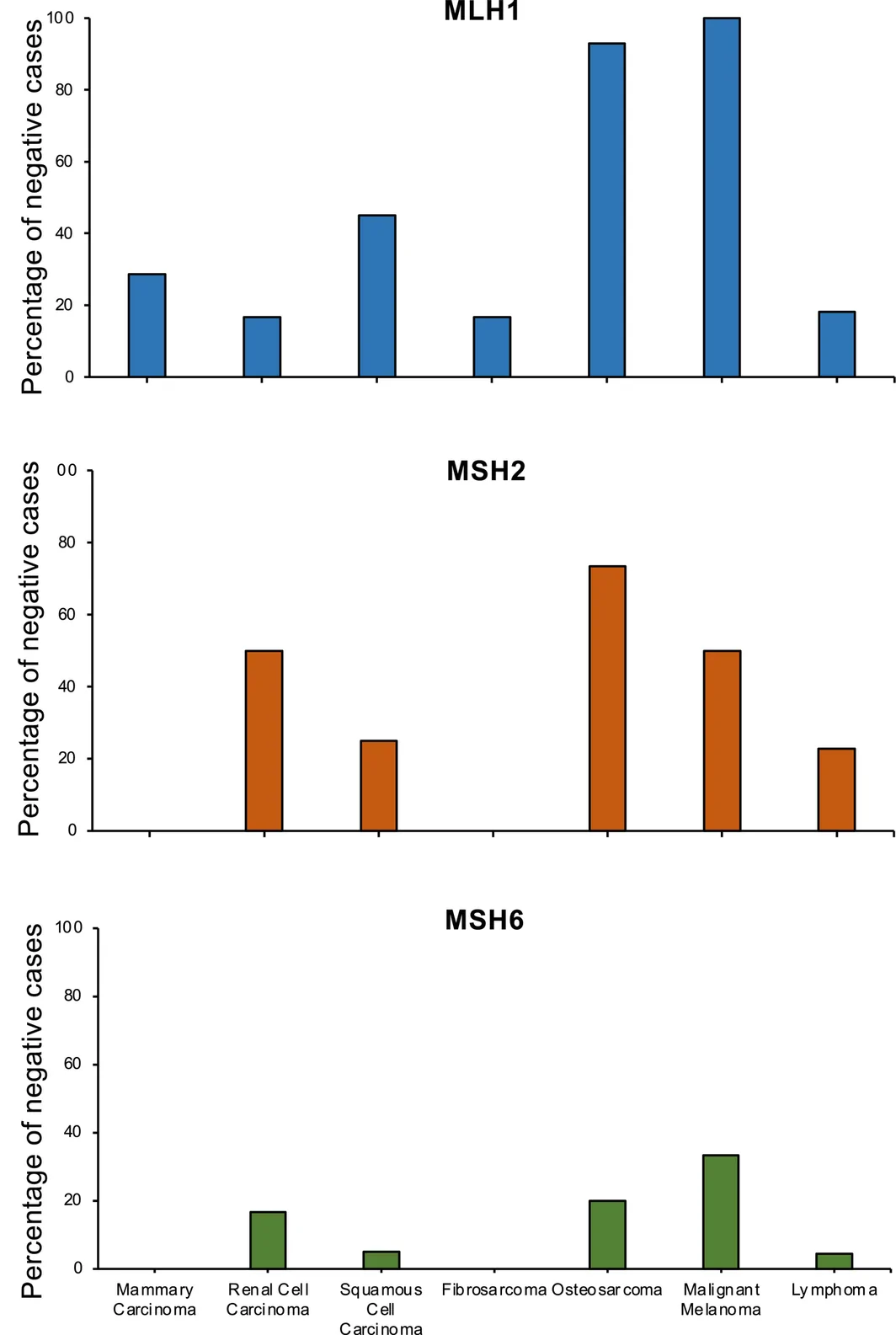
Frequency of MMR protein loss across various feline tumour types. The bar graphs show the percentage of cases negative for MLH1, MSH2 and MSH6 expression in each tumour type. MLH1 expression was lost in all melanoma cases (100%).

Cases with loss of two or more MMR proteins were detected in renal cell carcinomas (1/6), squamous cell carcinomas (5/20), osteosarcomas (11/15), melanomas (4/6) and lymphomas (4/22). Among these, complete loss of all three MMR proteins was observed in one renal cell carcinoma, one squamous cell carcinoma, three osteosarcomas and one melanoma case (Table 3). Representative micrographs demonstrating the complete loss of MLH1, MSH2 and MSH6 nuclear expression across these tumour types are provided in Supplemental Figure 3.

**TABLE 3:** Cases showing loss of two or more MMR proteins.

Tumour types	MLH1	MSH2	MSH6
Renal cell carcinoma (*n* = 1)	−	−	−
Squamous cell carcinoma (*n* = 5)	−	−	+
	−	−	+
	−	−	+
	−	−	−
	−	−	+
Osteosarcoma (*n* = 11)	−	−	+
	−	−	−
	−	−	−
	−	−	−
	−	−	+
	−	−	+
	−	−	+
	−	−	+
	−	−	+
	−	−	+
	−	−	+
Malignant melanoma (*n* = 4)	−	−	+
	−	−	+
	−	+	−
	−	−	−
Lymphoma (*n* = 4)	+	−	−
	−	−	+
	−	−	+
	−	−	+

*Note:* ‘−’ indicates loss/negative nuclear immunolabelling; “+” indicates retained nuclear immunolabelling.

### 3.3 Expression of MMR Proteins in Feline Cell Lines

Next, the expression of MLH1, MSH2 and MSH6 was analysed in various feline cell lines using the validated antibodies. All cell lines expressed the three MMR proteins; however, several feline tumour cell lines exhibited lower expression levels compared to the non‐neoplastic cell line fcwf‐4. Specifically, the mammary carcinoma cell lines FKNp, FONp and FONm and the B‐cell lymphoma line MS4 exhibited relatively low levels of MLH1 expression. Moreover, FKNp and FONp cells also showed reduced MSH2 and MSH6 expression. In addition, among the non‐neoplastic cell lines, the renal epithelial cell line CRFK displayed lower expression levels of all three MMR proteins than fcwf‐4 (Figure 4).

**FIGURE 4:**
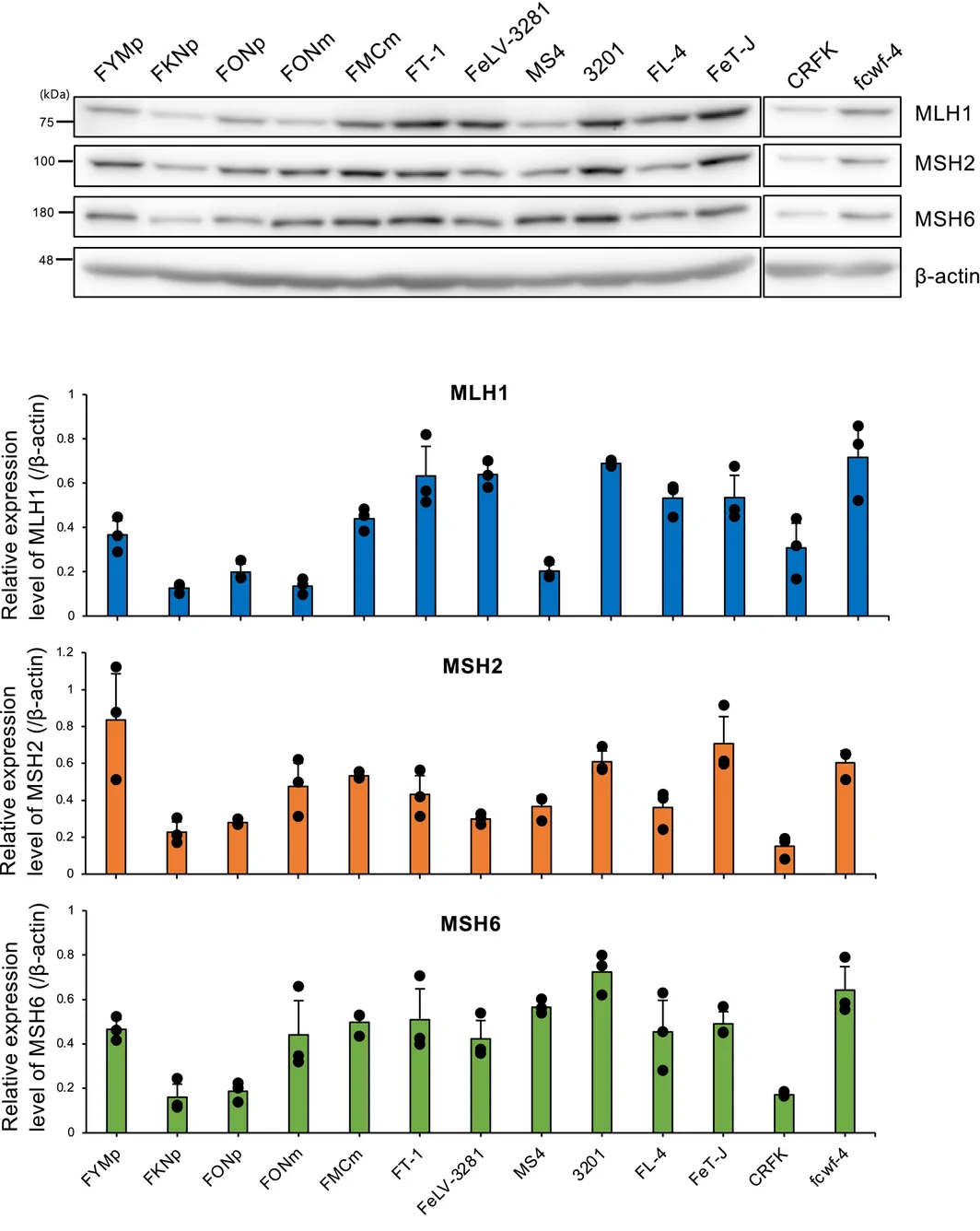
Basal expression of MMR proteins in feline cell lines. Western blot analysis of MLH1, MSH2 and MSH6 expression levels across a panel of feline neoplastic and non‐neoplastic cell lines (including CRFK and fcwf‐4). Proteins were separated with SDS‐PAGE, followed by western blotting with anti‐human MLH1 antibody, anti‐human MSH2 antibody or anti‐human MSH6 antibody. Comparison across the panel showed that several feline tumour cell lines exhibited lower MMR protein expression levels than the non‐neoplastic macrophage cell line fcwf‐4, whereas no cell lines showed a complete loss of any MMR protein. The relative expression of MLH1, MSH2 and MSH6 proteins was normalised to that of β‐actin based on the results of three independent experiments.

## 4 Discussion

This study establishes a practical IHC framework for evaluating DNA MMR protein expression in feline oncology. Commercially available anti‐human monoclonal antibodies against MLH1, MSH2 and MSH6 specifically detected the corresponding feline proteins, as demonstrated by loss of the relevant bands in CRISPR/Cas9‐engineered MMR‐deficient FYMp cell lines. The interdependence of the MutSα partners was also recapitulated: MSH6 was lost in MSH2‐deficient cells and MSH2 was reduced in MSH6‐deficient cells, consistent with the mutual‐stability relationship described in other species [19, 20, 21]. These data support the technical validity of cross‐reactive antibodies for profiling feline MMR by IHC.

Using these validated reagents, we observed decreased or absent nuclear staining of one or more MMR proteins across several tumour types. Because IHC is sensitive to pre‐analytic and analytic factors (fixation, block age, epitope accessibility, pigment or keratin interference, antibody clone/lot, retrieval and detection chemistry), an absent nuclear signal does not by itself prove loss of repair function. MLH1 loss was frequent in melanoma, osteosarcoma and squamous cell carcinoma. This pattern resembles human tumours in which MLH1 silencing is often linked to promoter hypermethylation [22, 23, 24], raising the possibility of similar mechanisms in feline neoplasms. These observations suggest that DNA‐repair abnormalities, potentially including an MSI–like phenotype, may occur in certain feline tumours. Given that dMMR/MSI‐H predicts response to immune checkpoint inhibitors in human oncology [6, 7, 25], MMR protein loss in cats warrants cautious exploration as a biomarker for future therapeutic studies.

The unexpectedly high frequency of MMR protein loss in melanoma, osteosarcoma and squamous cell carcinoma rather than those reported in humans and dogs [21, 26, 27, 28] deserves particular caution. Because IHC negativity can arise from technical factors, this unexpectedly high proportion of cases showing protein loss requires a structured differential: (i) residual technical influences despite our controls (e.g., pre‐analytical fixation variations or epitope accessibility); (ii) context‐dependent downregulation or stress‐induced suppression driven by inflammatory, hypoxic and oxidative microenvironments [29, 30, 31, 32], potentially modulated by feline‐specific metabolic and oxidative physiological traits [33, 34, 35]; and (iii) bona fide loss of MMR protein expression in a subset. Within this framework, feline gastrointestinal lymphoma showed largely preserved MMR protein expression in our series, consistent with a previous study [8], suggesting that frank dMMR is uncommon in that entity. To adjudicate biology versus artefact, we plan: (i) MLH1 promoter methylation assays in tumours with MLH1 loss; (ii) feline‐adapted MSI testing (PCR or NGS‐based); (iii) tumour mutational burden and mutational‐signature analysis to identify MMR‐defect patterns and (iv) contextual profiling of cooperating repair pathways (base‐excision, nucleotide‐excision and homologous recombination).

Melanocytic pigment is a known confounder in the interpretation of nuclear immunohistochemical staining, particularly when DAB‐based detection is used. To mitigate this issue, hydrogen peroxide–based melanin bleaching was applied to melanoma sections, and AEC detection was used instead of DAB. Because bleaching can attenuate antigenicity, we first validated that the same bleaching condition preserved nuclear immunoreactivity for MLH1, MSH2 and MSH6 in lymphoma tissues with previously confirmed positive staining. In melanoma samples, interpretation was therefore performed with particular attention to retained nuclear staining in adjacent non‐neoplastic elements within the same section, as defined in Section 2. Nevertheless, pigment‐related interference, bleaching‐associated antigen attenuation, fixation time, block age, necrosis, keratinisation and variable epitope accessibility cannot be fully excluded as residual sources of variability. Future studies should therefore include replication with alternative antibody clones or lots and, where appropriate, additional chromogenic systems such as alkaline phosphatase–based red detection to further minimise pigment‐related interpretive artefacts.

A further limitation is the inability to include PMS2 as a primary analyte. The anti‐human PMS2 clones tested did not yield nuclear staining in feline tissues under our conditions, precluding assessment of isolated PMS2 loss—a key feature in human dMMR algorithms. Optimisation of PMS2 detection is therefore a priority, and complementary MutS/MutL members such as MSH3, MLH3 and PMS1 may be informative in future feline panels [36, 37, 38]. Clinically, while human MSI‐H/dMMR tumours often benefit from PD‐1/PD‐L1 blockade, translation to cats will require a composite diagnostic framework. We propose a stepwise strategy in which IHC serves as an initial screen to nominate candidates for confirmatory testing and for prospective clinical evaluation with predefined biomarker endpoints [39].

In summary, we provide validated tools and initial landscape data for assessing MMR protein expression in feline tumours. Because PMS2 could not be reliably evaluated under the present IHC conditions, this MLH1/MSH2/MSH6 three protein panel should be regarded as an initial screening framework rather than a complete diagnostic surrogate for dMMR. The high rates of MMR protein loss observed in selected tumour types warrant cautious interpretation and immediate follow‐up with orthogonal assays and strengthened analytical controls. While individual cohort numbers are limited and functional confirmation is currently lacking, this foundational pilot study maps the baseline trends of MMR protein expression in feline cancers. Establishing dMMR in cats will likely require integrating IHC with MSI, methylation and genomic readouts before robust biological conclusions or therapeutic implications can be drawn.

## Author Contributions

**Shoma Nishibori** and **Takuya Mizuno:** conceptualisation. **Shoma Nishibori**, **Masashi Sakurai** and **Yumiko Kagawa:** data curation. **Kazuo Nishigaki** and **Takayuki Nakagawa:** resources. **Shoma Nishibori** and **Takuya Mizuno:** writing – original draft. **Masaya Igase** and **Takuya Mizuno:** supervision. All authors: writing – review and editing.

## Funding

The authors have nothing to report.

## Disclosure

We certify that the opinions expressed herein are only those of the authors.

## Consent

All owners signed informed‐consent forms to use each formalin‐fixed and paraffin‐embedded tissue specimen.

## Conflicts of Interest

The authors declare no conflicts of interest.

## Supporting information


**Table S1:** Clinical information on cats from which paraffin‐embedded tissue was collected.
**Figure S1:** Representative immunohistochemical staining of MLH1 in feline small intestinal lymphoma serving as an internal control validation.
**Figure S2:** Representative immunohistochemical staining of MLH1, MSH2 and MSH6 in a feline mammary tumour metastasised to a lymph node. Scale bars = 50 μm.
**Figure S3:** Representative micrographs of feline melanoma, osteosarcoma, squamous cell carcinoma and renal cell carcinoma exhibiting complete nuclear loss of MLH1, MSH2 and MSH6. Scale bars = 50 μm.

## Data Availability

The data that support the findings of this study are available from the corresponding author upon reasonable request.

## References

[1] D. Liu , G. Keijzers , and L. J. Rasmussen , “DNA Mismatch Repair and Its Many Roles in Eukaryotic Cells,” Mutation Research, Reviews in Mutation Research 773 (2017): 174–187, 10.1016/j.mrrev.2017.07.001. 28927527

[2] S. N. Thibodeau , G. Bren , and D. Schaid , “Microsatellite Instability in Cancer of the Proximal Colon,” Science 260 (1993): 816–819, 10.1126/science.8484122. 8484122

[3] N. Papadopoulos , N. C. Nicolaides , Y. F. Wei , et al., “Mutation of a mutL Homolog in Hereditary Colon Cancer,” Science 263 (1994): 1625–1629, 10.1126/science.8128251. 8128251

[4] A. Buckowitz , H.‐P. Knaebel , A. Benner , et al., “Microsatellite Instability in Colorectal Cancer Is Associated With Local Lymphocyte Infiltration and Low Frequency of Distant Metastases,” British Journal of Cancer 92 (2005): 1746–1753, 10.1038/sj.bjc.6602534. PMC2362037 15856045

[5] C. Luchini , F. Bibeau , M. J. L. Ligtenberg , et al., “ESMO Recommendations on Microsatellite Instability Testing for Immunotherapy in Cancer, and Its Relationship With PD‐1/PD‐L1 Expression and Tumour Mutational Burden: A Systematic Review‐Based Approach,” Annals of Oncology 30 (2019): 1232–1243, 10.1093/annonc/mdz116. 31056702

[6] D. T. Le , J. N. Uram , H. Wang , et al., “PD‐1 Blockade in Tumors With Mismatch‐Repair Deficiency,” New England Journal of Medicine 372 (2015): 2509–2520, 10.1056/NEJMoa1500596. PMC4481136 26028255

[7] D. T. Le , J. N. Durham , K. N. Smith , et al., “Mismatch Repair Deficiency Predicts Response of Solid Tumors to PD‐1 Blockade,” Science 357 (2017): 409–413, 10.1126/science.aan6733. PMC5576142 28596308

[8] D. Aberdein , J. S. Munday , L. Howe , A. F. French , and I. R. Gibson , “Widespread Mismatch Repair Expression in Feline Small Intestinal Lymphomas,” Journal of Comparative Pathology 147 (2012): 24–30, 10.1016/j.jcpa.2011.08.009. 22000951

[9] S. Nishibori , M. K. Kaneko , T. Nakagawa , et al., “Development of Anti‐Feline PD‐1 Antibody and Its Functional Analysis,” Scientific Reports 13 (2023): 6420, 10.1038/s41598-023-31543-6. PMC10126011 37095139

[10] S. Nishibori , Y. Takeda , M. Igase , and T. Mizuno , “Engineering an Fc‐Inert Feline IgG1 by Targeted Mutations: Application to Anti‐PD‐1 Antibody Development,” Veterinary Immunology and Immunopathology 288 (2025): 111000, 10.1016/j.vetimm.2025.111000. 40974651

[11] R. A. Crandell , C. G. Fabricant , and W. A. Nelson‐Rees , “Development, Characterization, and Viral Susceptibility of a Feline ( *Felis catus* ) Renal Cell Line (CRFK),” In Vitro 9 (1973): 176–185, 10.1007/bf02618435. 4130570

[12] N. C. Pedersen , J. F. Boyle , and K. Floyd , “Infection Studies in Kittens, Using Feline Infectious Peritonitis Virus Propagated in Cell Culture,” American Journal of Veterinary Research 42 (1981): 363–367. 6267959

[13] H. W. Snyder, Jr. , W. D. Hardy, Jr. , E. E. Zuckerman , and E. Fleissner , “Characterisation of a Tumour‐Specific Antigen on the Surface of Feline Lymphosarcoma Cells,” Nature 275 (1978): 656–658, 10.1038/275656a0. 212681

[14] T. Miura , H. Tsujimoto , M. Fukasawa , et al., “Structural Abnormality and Over‐Expression of the Myc Gene in Feline Leukemias,” International Journal of Cancer 40 (1987): 564–569, 10.1002/ijc.2910400422. 2822586

[15] H. Mochizuki , M. Takahashi , K. Nishigaki , et al., “Establishment of a Novel Feline Leukemia Virus (FeLV)‐Negative B‐Cell Cell Line From a Cat With B‐Cell Lymphoma,” Veterinary Immunology and Immunopathology 140 (2011): 307–311, 10.1016/j.vetimm.2010.12.010. 21272942

[16] J. K. Yamamoto , C. D. Ackley , H. Zochlinski , et al., “Development of IL‐2‐Independent Feline Lymphoid Cell Lines Chronically Infected With Feline Immunodeficiency Virus: Importance for Diagnostic Reagents and Vaccines,” Intervirology 32 (1991): 361–375, 10.1159/000150220. 1657826

[17] R. Kano , E. Sato , T. Okamura , S. Watanabe , and A. Hasegawa , “Expression of Bcl‐2 in Feline Lymphoma Cell Lines,” Veterinary Clinical Pathology 37 (2008): 57–60, 10.1111/j.1939-165X.2008.00013.x. 18366545

[18] R. Uyama , S.‐H. Hong , T. Nakagawa , et al., “Establishment and Characterization of Eight Feline Mammary Adenocarcinoma Cell Lines,” Journal of Veterinary Medical Science 67 (2005): 1273–1276, 10.1292/jvms.67.1273. 16397390

[19] J. T. Drummond , G. M. Li , M. J. Longley , and P. Modrich , “Isolation of an hMSH2‐p160 Heterodimer That Restores DNA Mismatch Repair to Tumor Cells,” Science 268 (1995): 1909–1912, 10.1126/science.7604264. 7604264

[20] P. Zhao , L. Li , X. Jiang , and Q. Li , “Mismatch Repair Deficiency/Microsatellite Instability‐High as a Predictor for Anti‐PD‐1/PD‐L1 Immunotherapy Efficacy,” Journal of Hematology & Oncology 12 (2019): 54, 10.1186/s13045-019-0738-1. PMC6544911 31151482

[21] S. Inanaga , M. Igase , Y. Sakai , et al., “Mismatch Repair Deficiency in Canine Neoplasms,” Veterinary Pathology 58 (2021): 1058–1063, 10.1177/03009858211022704. 34538134

[22] J. G. Herman , A. Umar , K. Polyak , et al., “Incidence and Functional Consequences of hMLH1 Promoter Hypermethylation in Colorectal Carcinoma,” Proceedings of the National Academy of Sciences of the United States of America 95 (1998): 6870–6875, 10.1073/pnas.95.12.6870. PMC22665 9618505

[23] H. M. Tawfik , N. M. R. A. El‐Maqsoud , B. H. A. A. Hak , and Y. M. El‐Sherbiny , “Head and Neck Squamous Cell Carcinoma: Mismatch Repair Immunohistochemistry and Promoter Hypermethylation of hMLH1 Gene,” American Journal of Otolaryngology 32 (2011): 528–536, 10.1016/j.amjoto.2010.11.005. 21353335

[24] L. Pergoli , C. Favero , R. M. Pfeiffer , et al., “Blood DNA Methylation, Nevi Number, and the Risk of Melanoma,” Melanoma Research 24 (2014): 480–487, 10.1097/CMR.0000000000000112. PMC6857929 25026000

[25] T. Landre and G. Des Guetz , “Microsatellite Instability‐High Status as a Pan‐Cancer Biomarker for Immunotherapy Efficacy,” Cancer Immunology, Immunotherapy 74 (2025): 122, 10.1007/s00262-025-03980-x. PMC11861458 39998698

[26] K. Vasan , S. Anand , L. Satgunaseelan , et al., “Mismatch Repair Protein Loss in Cutaneous Head and Neck Squamous Cell Carcinoma,” Journal of Surgical Oncology 122 (2020): 1755–1760, 10.1002/jso.26218. 32926758

[27] S. W. Lam , M. Kostine , N. F. C. C. de Miranda , et al., “Mismatch Repair Deficiency Is Rare in Bone and Soft Tissue Tumors,” Histopathology 79 (2021): 509–520, 10.1111/his.14377. PMC8518745 33825202

[28] T. Gambichler , C. Finis , N. Abu Rached , et al., “Expression of DNA Mismatch Repair Proteins in Melanoma Patients Treated With Immune Checkpoint Inhibitors,” Journal of Cancer Research and Clinical Oncology 149 (2023): 1241–1247, 10.1007/s00432-022-04002-4. PMC9984342 35419731

[29] A. Kondo , R. Safaei , M. Mishima , H. Niedner , X. Lin , and S. B. Howell , “Hypoxia‐Induced Enrichment and Mutagenesis of Cells That Have Lost DNA Mismatch Repair,” Cancer Research 61 (2001): 7603–7607. 11606400

[30] C. L. Chang , G. Marra , D. P. Chauhan , et al., “Oxidative Stress Inactivates the Human DNA Mismatch Repair System,” American Journal of Physiology. Cell Physiology 283 (2002): C148–C154, 10.1152/ajpcell.00422.2001. 12055083

[31] V. T. Mihaylova , R. S. Bindra , J. Yuan , et al., “Decreased Expression of the DNA Mismatch Repair Gene Mlh1 Under Hypoxic Stress in Mammalian Cells,” Molecular and Cellular Biology 23 (2003): 3265–3273, 10.1128/MCB.23.9.3265-3273.2003. PMC153206 12697826

[32] N. Valeri , P. Gasparini , M. Fabbri , et al., “Modulation of Mismatch Repair and Genomic Stability by miR‐155,” Proceedings of the National Academy of Sciences of the United States of America 107 (2010): 6982–6987, 10.1073/pnas.1002472107. PMC2872463 20351277

[33] J. E. Bauer , “Metabolic Basis for the Essential Nature of Fatty Acids and the Unique Dietary Fatty Acid Requirements of Cats,” Journal of the American Veterinary Medical Association 229 (2006): 1729–1732, 10.2460/javma.229.11.1729. 17144816

[34] M. Hoenig , N. Pach , K. Thomaseth , A. Le , D. Schaeffer , and D. C. Ferguson , “Cats Differ From Other Species in Their Cytokine and Antioxidant Enzyme Response When Developing Obesity,” Obesity 21 (2013): E407–E414, 10.1002/oby.20306. 23408676

[35] M. H. Court , “Feline Drug Metabolism and Disposition: Pharmacokinetic Evidence for Species Differences and Molecular Mechanisms,” Veterinary Clinics of North America. Small Animal Practice 43 (2013): 1039–1054, 10.1016/j.cvsm.2013.05.002. PMC3811070 23890237

[36] W. Edelmann , A. Umar , K. Yang , et al., “The DNA Mismatch Repair Genes Msh3 and Msh6 Cooperate in Intestinal Tumor Suppression,” Cancer Research 60 (2000): 803–807. 10706084

[37] A. Srivatsan , N. Bowen , and R. D. Kolodner , “Mispair‐Specific Recruitment of the Mlh1‐Pms1 Complex Identifies Repair Substrates of the *Saccharomyces cerevisiae* Msh2‐Msh3 Complex,” Journal of Biological Chemistry 289 (2014): 9352–9364, 10.1074/jbc.M114.552190. PMC3979400 24550389

[38] C. M. Furman , R. Elbashir , and E. Alani , “Expanded Roles for the MutL Family of DNA Mismatch Repair Proteins,” Yeast 38 (2020): 39–53, 10.1002/yea.3512. PMC7854849 32652606

[39] K. L. Limpose , A. H. Corbett , and P. W. Doetsch , “BERing the Burden of Damage: Pathway Crosstalk and Posttranslational Modification of Base Excision Repair Proteins Regulate DNA Damage Management,” DNA Repair 56 (2017): 51–64, 10.1016/j.dnarep.2017.06.007. PMC5576989 28629773

