# Supplementary material for: Immunohistochemical Characterisation of Mismatch Repair Proteins in Feline Cancers: A Pilot Study Using Validated Cross‐Reactive Antibodies
Source: Vet Comp Oncol. 2026 Aug 16;24(4):809–18. doi: 10.1111/vco.70105 (PMC13745631; doi:10.1111/vco.70105)

Supplemental Table 1. Clinical information on cats from which paraffin-embedded tissue was collected.

Diagnosis	Breed	Age (year-old)	Sex	Tumor site of origin	Remarks
Mammary Carcinoma	Mixed	7	FS	Left M1 and M2	Metastasis to the left collateral axillary lymph node
	Mixed	8	FI	Right	Metastasis to the right axillary lymph node
	Mixed	8	FS	Right M2	
	Abyssinian	11	FS	Left M3 and M4	
	Mixed	12	FS	Right M3	Metastasis to the right inguinal lymph node
	Mixed	13	FI	Left M1 and M2	
	Mixed	15	FI	Right M3 and M4	Metastasis to the left inguinal and accessory inguinal lymph nodes
Renal Cell Carcinoma	Mixed	4	FI	Left kidney	
	Mixed	12	FS	Right kidney	
	Mixed	13	FS	Right kidney	
	Mixed	14	MN	Right kidney	
	UN	UN	UN	Right kidney	
	Mixed	UN	MN	Right kidney	
Squamous Cell Carcinoma	Mixed	3	FS	Tongue	
	Mixed	7	FS	Tongue	
	Norwegian forest cat	9	FS	Tongue	
	Mixed	9	MN	Mandible	
	Mixed	11	MN	Tongue	
	Mixed	11	FS	Mandible	
	Japanese domestic cat	11	MN	Mandible	
	Japanese domestic cat	12	FS	Mandible	
	Japanese domestic cat	12	MN	Tongue	
	Mixed	12	MN	Tongue	
	Mixed	13	FS	Tongue	
	Mixed	14	FS	Nasal philtrum	
	Chinchilla	14	FS	Tongue	
	Chinchilla	14	FS	Intraoral	
	American short hair	14	FS	Mandible	
	Mixed	14	FS	Mandible	
	Japanese domestic cat	15	FS	Tongue	
	Mixed	15	FS	Mandible	
	Mixed	17	FI	Nasal philtrum	
	Mixed	UN	UN	Left pinna	Surface ulceration
Fibrosarcoma	Maine Coon	7	FS	Subcutaneous between the shoulders (Recurrent)	
	Norwegian forest cat	8	MN	Right abdominal subcutaneous	
	Mixed	10	MN	Scapula	Partial chondroid differentiation
	Mixed	10	FS	Subcutaneous between the shoulders	Infiltration and proliferation within the muscle layer
	Mixed	12	FS	Left subcutaneous tarsus	
	Mixed	12	MN	Right subcutaneous tarsus	
Osteosarcoma	Mixed	2	FS	Femur	Metastasis to the lung
	Mixed	8	MN	Humerus	Metastasis to the left parasternum and left ilium
	Norwegian Forest Cat	9	FS	Radius	Metastasis to the lung
	Mixed	10	FI	Radius and Ulna	
	American short hair	10	FS	Femur	
	Mixed	10	MN	Scapula	
	American short hair	12	FS	Tibia	Metastasis to the skin and lung
	Mixed	12	MN	Humerus	
	Mixed	12	MI	Humerus	Metastasis to the lung
	Mixed	12	FS	Radius and Ulna	
	American short hair	13	FI	Femur	
	Mixed	16	FS	Femur	
	American short hair	17	FS	Tibia	
	Mixed	UN	MN	Radius	
	Mixed	UN	MN	Femur and Tibia	Metastasis to the contralateral knee
Malignant Melanoma	Mixed	5	FS	Right metacarpal pad	Pigmented melanoma; surface ulceration present
	Mixed	8	FS	Right metatarsal pad	Pigmented melanoma
	Mixed	10	FS	Left eyelid (Recurrent)	Pigmented melanoma
	Mixed	11	MN	Left hindlimb (Recurrent)	Pigmented melanoma
	UN	23	FS	Neck	Pigmented melanoma
	Mixed	23	FS	Lower jaw (Recurrent)	Amelanotic melanoma
Lymphoma	Mixed	0	UN	Small intestine	Large cell lymphoma
	Mixed	4	MN	Small intestine	Large cell lymphoma
	Mixed	4	FS	Intestinal tract	Large cell lymphoma
	Mixed	4	MN	Small intestine	Large cell lymphoma
	Mixed	6	MN	Ileocecum	Large cell lymphoma
	Mixed	7	FS	Iliocecum	Large cell lymphoma
	UN	9	MI	Intestinal tract	Large granular lymphoma
	Maine Coon	9	MN	Right thigh skin	
	Mixed	11	FS	Small intestine	Large cell lymphoma
	Mixed	11	FS	Intestinal tract	Large granular lymphoma
	Mixed	11	FS	Duodenum	Small cell lymphoma
	UN	11	MI	Duodenum	Small cell lymphoma
	Mixed	13	FS	Left pinna	Large T-cell lymphoma
	Mixed	14	FS	Jejunum	Large cell lymphoma
	Mixed	14	MN	Jejunum	Large granular lymphoma
	Mixed	15	FS	Duodenum	Small cell lymphoma
	Mixed	15	FS	Duodenum, gastric, pancreas	Small cell lymphoma; pancreatitis chronic
	Mixed	15	MN	Caecum	Large cell lymphoma
	Mixed	15	MN	Subcutaneous Mass in the back	B-cell lymphoma
	Mixed	16	MN	Duodenum	Large cell lymphoma
	Mixed	17	FS	Ilium, caecum	Large cell lymphoma
	Mixed	17	MN	Tarsal skin	Large B-cell lymphoma

UN, unknown; FS, female spayed; FI, female intact; MN, male neutered; MI, male intact.
M1–M4 indicate cranial thoracic, caudal thoracic, cranial abdominal, and caudal abdominal mammary glands, respectively.

Supplemental Figure. 1

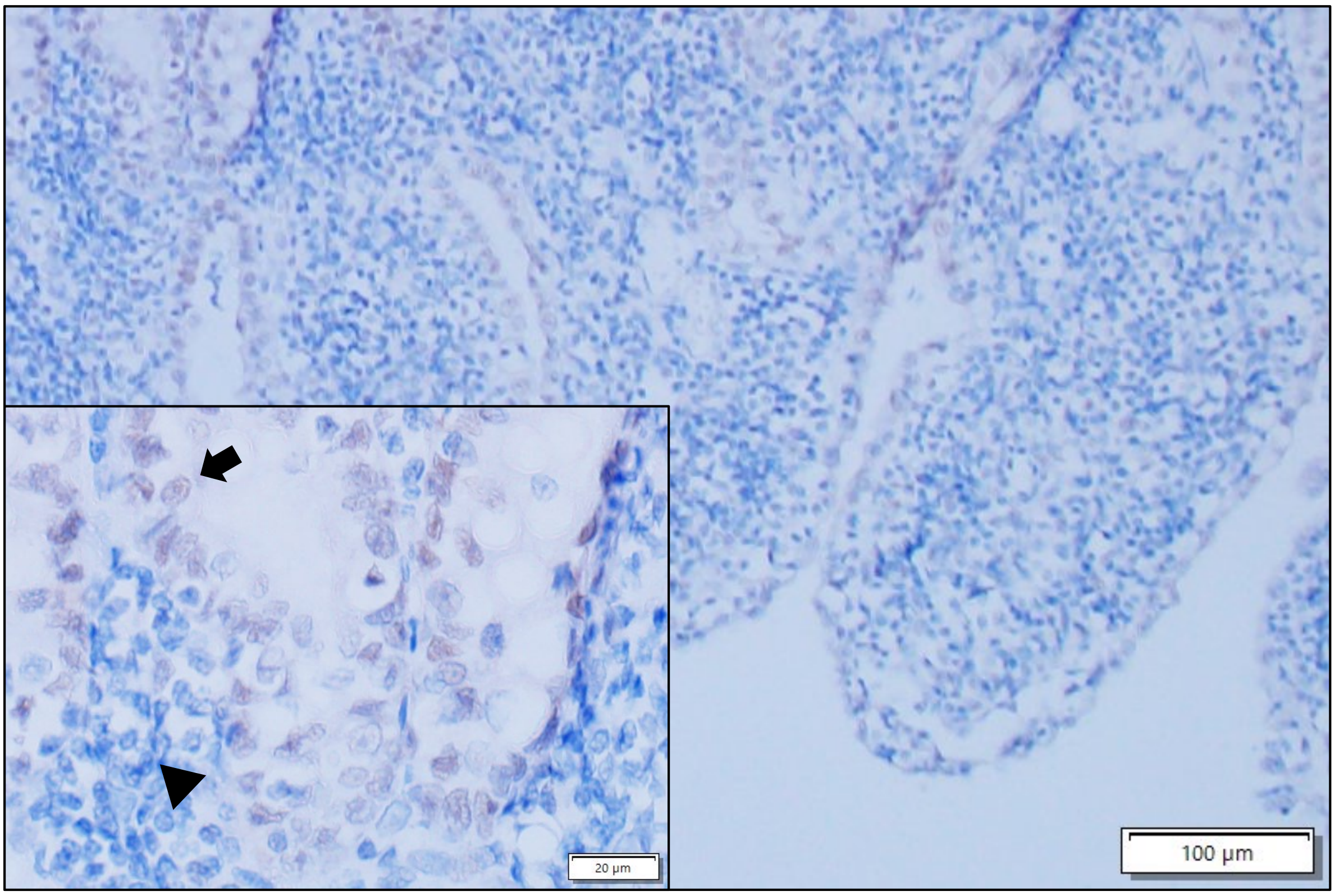

Supplemental Figure. 2

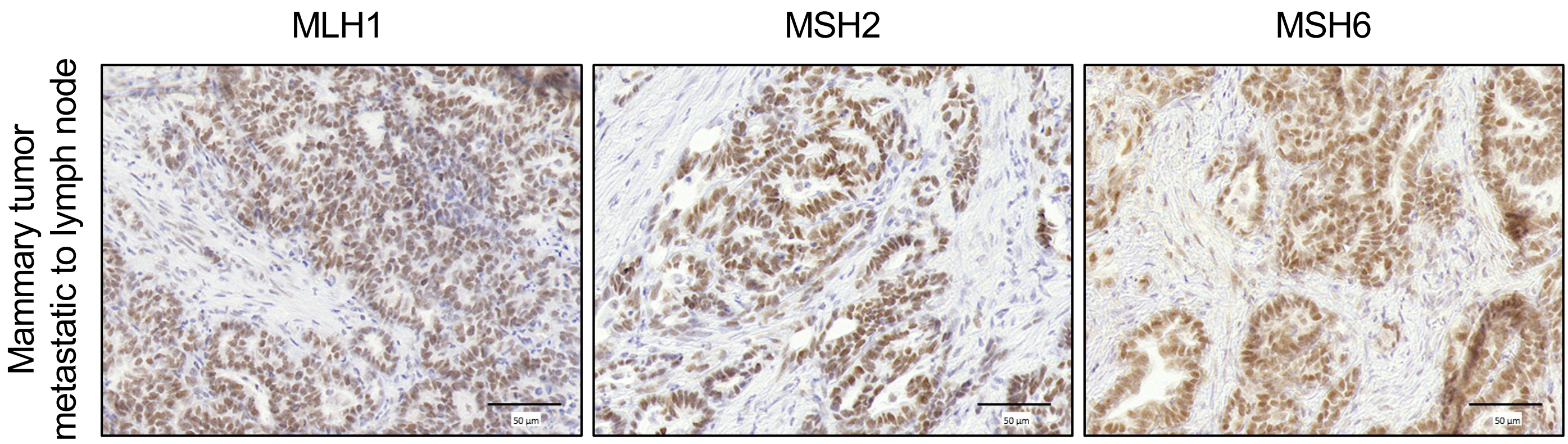

Supplemental Figure. 3

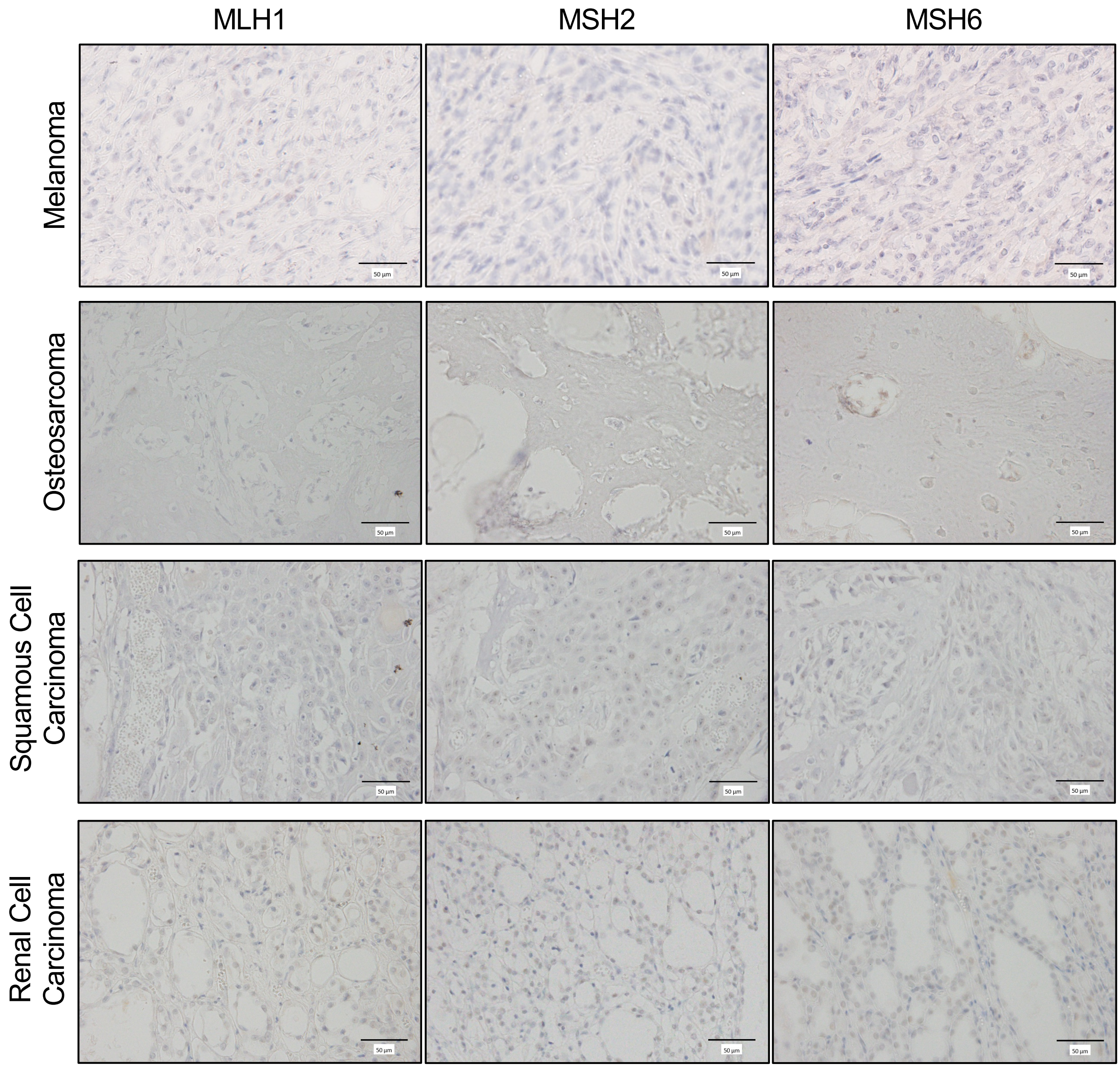

Supplement: Supplementary file 1 [file VCO-24-809-s001.pdf]
